# Emerging therapies in idiopathic inflammatory myopathies

**DOI:** 10.1177/22143602251384098

**Published:** 2025-10-23

**Authors:** Merve Kocyigit, Jan Piet van Hamburg, Eleonora Aronica, Anneke J van der Kooi, Sander W Tas, Joost Raaphorst

**Affiliations:** 1 Department of Neurology, Amsterdam Neuroscience, Amsterdam University Medical Center, 26066 University of Amsterdam, Amsterdam, the Netherlands; 2 Department of Rheumatology and Clinical Immunology, Amsterdam Rheumatology and Immunology Centre, Amsterdam University Medical Center, University of Amsterdam, Amsterdam, the Netherlands; 3 Department of Experimental Immunology, Amsterdam University Medical Center, University of Amsterdam, Amsterdam, the Netherlands; 4 Department of Neuropathology, Amsterdam Neuroscience, Amsterdam University Medical Center, 26066 University of Amsterdam, Amsterdam, the Netherlands

**Keywords:** idiopathic inflammatory myopathy, myositis, treatment, emerging therapies, pathogenesis

## Abstract

Idiopathic inflammatory myopathies (IIM), also known as myositis, are a group of heterogeneous autoimmune diseases characterized by muscle inflammation and frequent involvement of extramuscular organs. Autoantibodies are present in approximately 70% of the patients. Despite treatment advances, management of IIM largely relies on empirical approaches and current treatment options lack robust evidence, with the exception of intravenous immunoglobulin (IVIg). Even with immunosuppressive therapy, up to 80% of the patients continue to experience ongoing disease activity, functional impairment and a significant reduced quality of life. Several challenges complicate the management of IIM, including rare occurrence, heterogeneity of the disease, systemic manifestations, higher prevalence of malignancies and the complexity of conducting large-scale clinical trials in rare diseases. Along with this, therapeutic development has long been hindered by an incomplete understanding of the disease pathogenesis. However, recent insights into the molecular and cellular mechanisms of IIM have revealed novel therapeutic targets. In this review, we will discuss the underlying immunopathogenesis of IIM, including the role of potentially pathogenic autoantibodies, B and T cells, the interferon (IFN) pathway and the complement system. We will also review current treatment strategies and provide an overview of emerging and promising new treatments tested in recently published clinical trials or ongoing clinical trials, including chimeric antigen receptor (CAR) T-cell therapy, T-cell engagers, T-cell targeting therapies, neonatal Fc receptor (FcRn) inhibitors, and small-molecule inhibitors targeting intracellular signaling molecules such as Janus kinases (JAKs), IFNs, and complement.

## Introduction

Idiopathic inflammatory myopathies (IIM), commonly known as myositis, are a group of autoimmune diseases characterized by muscle inflammation with heterogeneous clinical manifestations, frequently involving other organ systems including skin, lungs, joints and heart. IIM can be divided into different subtypes: dermatomyositis (DM), immune-mediated necrotizing myopathy (IMNM), antisynthetase syndrome (ASyS), overlap myositis (OM), non-specific myositis and inclusion body myositis (IBM).^1–3^ Polymyositis (PM) is a debated subtype and has largely been reclassified into more distinct subgroups as IMNM, ASyS and IBM in the last two decades, making it the rarest IIM subtype currently.^3–5^ Although clinical and serological features vary among the different subtypes, common symptoms include muscle weakness and muscle pain. Autoantibodies are found in 60–70% of the patients, which are either myositis-specific antibodies (MSA) or myositis-associated antibodies (MAAs).^6–8^ MSAs are associated with distinct subgroups and unique clinical phenotypes of IIM and have an increasingly important diagnostic and prognostic value. MAAs can be found in both OM and in connective tissue diseases (CTD) and are not specific for IIM.^6,9^

DM is characterized by muscle weakness in combination with typical cutaneous manifestations. Approximately 70% of the patients test positive for MSAs (anti-MDA5, anti-NXP2, anti-TIF1y, anti-Mi2, anti-SAE).^10^ ASyS patients have autoantibodies against aminoacyl transfer RNA (tRNA) synthetases by definition and exhibit one or more of the following clinical manifestations: myositis, interstitial lung disease (ILD), rash and/or arthritis.^11,12^ The most prevalent autoantibodies in ASyS are anti-Jo-1 (72%), anti-PL7 (11.5%) and anti-PL12 (10%).^11^ OM occurs in coexistence of a CTD with different combinations of arthritis, Raynaud's phenomenon, ILD and scleroderma; MAAs are present including anti-PM/Scl, anti-Ku, anti-U1RNP.^13,14^ Patients with IMNM typically have rapidly developing severe symmetrical muscle weakness and extremely high serum creatine kinase levels. Approximately 60–80% of IMNM patients have autoantibodies, either anti-SRP or anti-HMGCR.^15^ Of note, atypical phenotypes of IMNM with non-classical patterns of muscle weakness, isolated high CK levels or a slower disease progress are increasingly recognized in particularly seropositive IMNM patients.^16^ IBM represents a distinct entity among IIM as this subtype has a slowly progressive disease course with gradual onset and is refractory to immunosuppressive treatment, therefore it is often referred as non-treatable IIM.^17^ IBM is characterized by slowly progressive asymmetrical muscle weakness, including finger flexor and quadriceps weakness often in combination with dysphagia. Anti-cN1A antibodies are present in 33–76% of the patients.^17,18^

Despite immunosuppressive treatment, up to 80% of patients with treatable IIM have chronic disease activity and significant functional disability with reduced quality of life.^19–22^ Patients experience a high disease burden, with a threefold increase in mortality compared to the general population.^1,19,20,22,23^ Insufficiently controlled myositis with persistent inflammation can result in significant and irreversible muscle damage, as well as damage to extramuscular organs such as ILD, leading to substantial morbidity and mortality.^21^ Furthermore, current treatments are associated with significant and often undesirable side effects, particularly the long-term adverse effects of steroids. Altogether, there is a high unmet need for new treatments.

Until recently, developing novel therapeutics for IIM has been challenging due to poor understanding of disease pathogenesis and complexities with performing large clinical trials in IIM. However, in recent years, major advancements in understanding the molecular and cellular pathways contributing to the pathogenesis of IIM have led to the development of new targeted treatments that may improve efficacy and tolerability. In this review, we will provide an overview of insights into immunopathology and emerging therapies in IIM, including chimeric antigen receptor (CAR) T-cell therapy, T-cell engager (TCE) therapy, T-cell targeting therapy, neonatal Fc receptor (FcRn) blockers and inhibitors of the Janus kinase/signal transducer and activator of transcription (JAK/STAT) pathway, interferon (IFN) pathway and complement system.

## Immunopathogenesis of IIM

**Figure 1.**
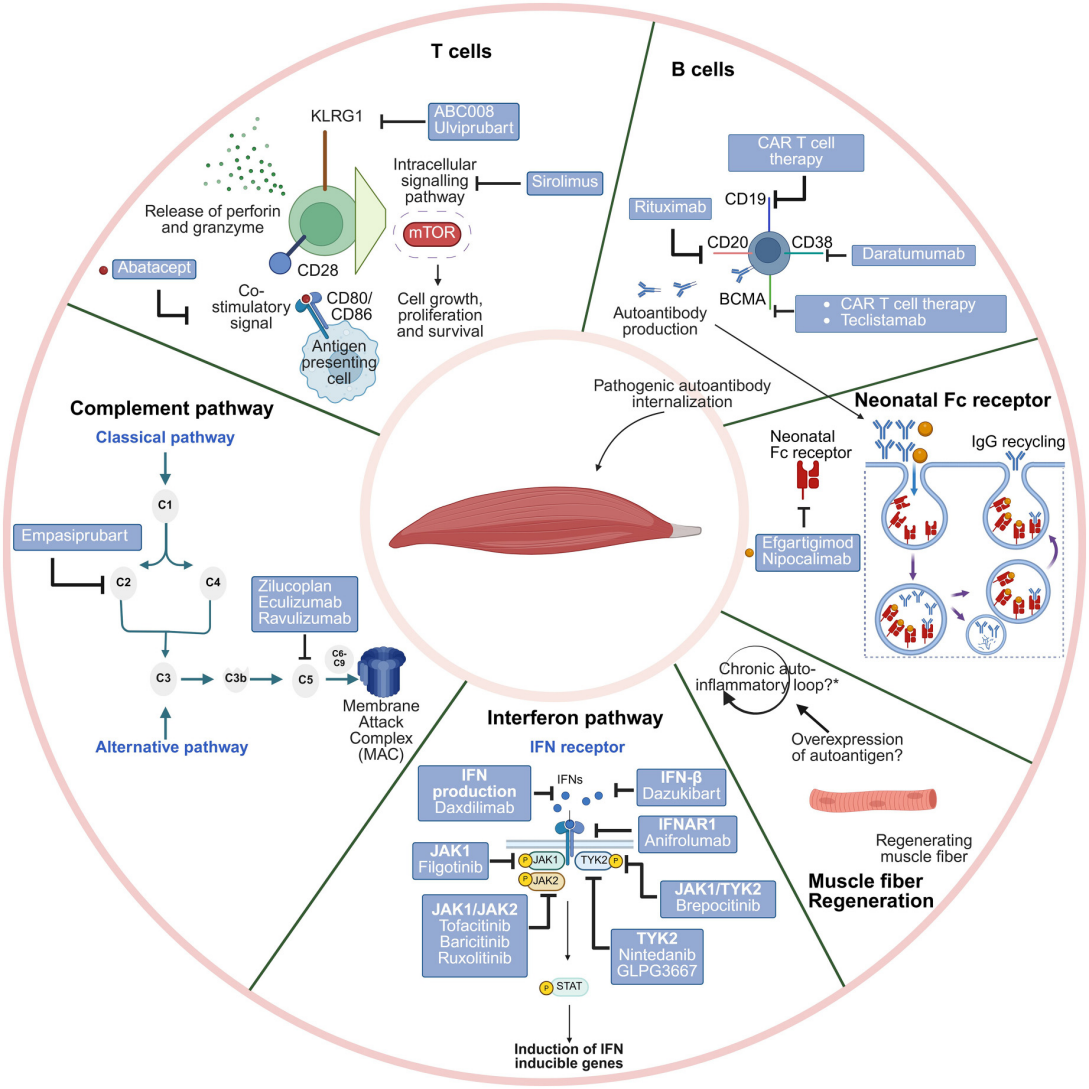
Immunopathogenesis of IIM: Molecular and cellular pathways and therapeutic targets.

Legend: The figure focuses on new and emerging therapies and related immunological pathways in idiopathic inflammatory myopathies (IIM). The figure is not intended to provide an exhaustive overview of all existing treatment options and related pathways in IIM. Tumor necrosis factor—alpha (TNF-α) inhibitors (e.g., infliximab) and B-cell activating factor (BAFF) inhibitors—(e.g., belimumab)—are left out; previous trials with these agents were not conclusive or negative. * It is hypothesized by some authors that regenerating muscle fibers are immunogenic (express autoantigens, which attract autoantibodies), potentially leading to a chronic autoinflammatory loop.

The pathophysiology of IIM is complex and only partially understood, involving dysregulation across various components of the immune system (Figure 1). Compared to other IIM subtypes, IBM exhibits a distinct disease course and phenotype and is refractory to immunosuppressive treatments. These findings imply a different pathogenesis of IBM. The pathogenesis of IBM is discussed in this paragraph and in separate paragraphs below regarding the role of immune cells, in more detail. The pathogenesis of non-IBM IIM is discussed in separate paragraphs (on autoantibodies, B cells, T cells, the IFN pathway and the complement system (Figure 1)). Both autoimmune and degenerative mechanisms are implicated in IBM, and which mechanism is the primary driver of disease pathogenesis remains a subject of ongoing debate. However, several lines of evidence in recent years suggest that inflammatory mechanisms precede the degenerative components.^24^ The hypothesis that IBM has a degenerative component is supported by disease onset after 45 years of age and the presence of protein deposits, such as p62, β-​amyloid and TDP43, in muscle tissue which are associated with several other neurodegenerative diseases.^24–26^ Another hypothesis proposes IBM as a spectrum disease and that PM with mitochondrial abnormalities (PM-mito) may represent early stages of IBM.^27–30^ In this line, similarities in mitochondrial dysfunction in IBM and PM-mito have been found.^31^ Evidence for autoimmunity in IBM is substantiated by the presence of autoantibodies and autoantibody-secreting plasma cells in muscle tissue; These will be further addressed below in the paragraph role of B cells in IIM.^32–34^

In recent years, significant progress has been made in unraveling the immunological mechanisms underlying IIM, paving the way for more targeted therapeutic strategies.

### Pathogenicity of autoantibodies

The question whether MSAs are pathogenic or merely an epiphenomenon has been addressed by several authors providing strong evidence that certain MSAs contribute to disease pathogenesis.^35,36^ Table 1 provides an overview of the evidence supporting the pathogenicity of certain MSAs and MAAs. Clinical observations supporting the pathogenic role of MSAs, such as anti-Jo-1, anti-TIF1γ, anti-Mi2, anti-MDA5, anti-SRP and anti-HMGCR include: (1) a correlation between serum autoantibody levels and disease activity, serological markers or muscle weakness; (2) clinical remission in patients treated with therapies that reduce autoantibody levels, such as rituximab; and (3) a clear association of specific autoantibodies with distinct clinical phenotypes.^9,35,37–41^

**Table 1.** Summary of evidence for supporting potential pathogenicity of autoantibodies in idiopathic inflammatory myopathies.

Autoantibody	Clinical pathogenicity criteria				Experimental pathogenicity criteria			
	Specific for IIM (subtype)	Present before disease onset	Correlation of autoantibody levels and disease activity	Improvement in disease activity with reducing autoantibody	Inducing disease symptoms with transplacental transfer	Inducing disease symptoms with transfer to mice	Immunization leading to production of autoantibodies and similar disease process	Autoantibody is found along with target antigen at the site of tissue damage
**Anti-HMGCR**	+ (IMNM)	ND	+	+	ND	+	+	+
**Anti-SRP**	+ (IMNM)	ND	+	+	ND	+	+	+
**Anti-Jo-1**	+ (ASyS)	ND	+	+	ND	ND	Immunization with derivatives of HISrS led to disease symptoms	+
**Anti-Mi2**	+ (DM)	ND	+	+	ND	ND	ND	+
**Anti-MDA5**	+ (DM)	ND	+	+	ND	ND	Circumstantial evidence ^(REF: 35)^	+
**Anti-Pm/Scl**	-	ND	ND	-	ND	ND	ND	+

Legend: autoantibodies (myositis-specific autoantibodies (MSAs) and myositis-associated autoantibodies (MAAs)) that have at least 1 experimental criterion of pathogenicity (described in text) are included in the table. +: yes; -: no; ND: not demonstrated; Anti-HMGCR: anti-3-hydroxy-3-methylglutaryl-co-enzym A reductase; Anti-Jo-1: anti-histidyl-transfer-RNA-synthetase; Anti-MDA5: anti-melanoma differentiation-associated protein 5; Anti-Mi2: anti-nucleosome remodeling deacetylase complex (Mi-2); Anti-Pm/Scl: anti-Polymyositis/scleroderma ; Anti-SRP: anti-signal recognition particle; Anti-TIF1γ: anti-transcription intermediary factor 1-gamma; ASyS: anti-synthetasesyndrome; DM: dermatomyositis; HisRS: histidyl-transfer RNA-synthetase; IMNM: immune-mediated necrotizing myopathy; OM: overlapmyositis.

For some of the autoantibodies, experimental data also support a direct pathogenic role. For example, an *in vitro* study demonstrated that serum from anti-SRP–positive IMNM patients reduced the survival of human myoblasts.^42^ Another study showed that purified anti-SRP and purified anti-HMGCR autoantibodies induced muscle fiber atrophy as well as reduced myoblast fusion *in vitro*, indicating that IMNM autoantibodies may impair muscle fiber regeneration.^43^

*In vivo* studies in mouse models provide further support for the pathogenic role of IMNM autoantibodies as passive transfer of patient-derived anti-SRP and anti-HMGCR autoantibodies led to muscle weakness and myofiber necrosis.^44^ Similarly, active immunization with SRP and HMGCR antigens in mice resulted in the production of anti-SRP and anti-HMGCR autoantibodies, and a subsequent decrease in muscle strength.

Recent *in vitro* studies suggest a pathogenic role for other MSAs: anti-Mi2, anti-Jo-1, anti-PM/Scl, and anti-MDA5 accumulate in the muscle fibers within the same cellular compartment as their target antigen, where they interfere with their function.^45^ Muscle biopsies from IIM patients with anti-Mi2 or anti-PM/Scl antibodies showed distinct gene expression patterns consistent with dysfunction of their respective target antigens.^45^ For other autoantibodies, a less distinct gene expression signature has been observed. However, indirect evidence, including overexpression of type I IFN-inducible genes, strongly suggests that these autoantibodies interfere with the function of their target antigen.^45^ Furthermore, the addition of specific autoantibodies to cultured muscle cells induced gene expression changes similar to those seen in patient muscle biopsies. These findings indicate the ability of MSAs for reaching their target tissue antigen and impairing their function. However, two intriguing key questions remain: (1) how do these autoantibodies enter muscle cells to access their intracellular targets, given that antibodies typically exert their effector functions in blood and other tissues without entering cells? In other autoimmune diseases, for example systemic lupus erythematosus (SLE), where antibodies target intracellular antigens such as anti-double stranded DNA (anti-dsDNA), several mechanisms have been proposed including an aberrant immune response to apoptotic cells but not antibody internalization.^46–48^ A role for surface Fc receptors in mediating the penetration of autoantibodies into living cells has been described in mixed connective tissue disease.^49^ However, skeletal muscle fibers usually do not express Fc receptors. An alternative hypothesis is that minor damage to the cell membrane—possibly due to a viral infection, or mechanical stress from muscle contraction or unknown factors —may facilitate the entry of autoantibodies into muscle cells. Consequently, the underlying mechanisms leading to autoantibody internalization in muscle tissue and how this potentially results in muscle dysfunction remain to be elucidated.^45,50^ (2) Another key question is why only muscles and other specific organs such as lung and skin are targeted, as autoantigens are present in each cell. One hypothesis for this is that autoantigens are differentially expressed in these target cells.^35^ Altogether, several central questions regarding mechanisms of autoantibody internalization and through which mechanism they possibly result in selective organ damage in IIM have to be answered.

Several experimental studies further support the pathogenic role of specific autoantibodies in IIM. For example immunization of mice with murine derivatives of histidyl-tRNA synthetase led to inflammatory muscle and lung disease, closely resembling ASyS.^51,52^ Similarly, immunization of mice with TIF1γ induced experimental myositis with muscle fiber necrosis and atrophy.^53^ More recently, serum from newly diagnosed IIM patients was shown to impair force generation in skeletal muscle fibers, although this effect was not observed when isolated IgG was applied alone.^54^ By contrast, the introduction of purified IgGs from anti-SRP and anti-HMGCR patients into a three-dimensional (3D) skeletal muscle cell culture model has demonstrated distinct pathogenic effects, with anti-SRP IgGs inducing muscle atrophy and anti-HMGCR IgGs leading to reduced contractile force.^55^ These conflicting findings highlight potentially diverse pathogenic effects of autoantibodies in IIM and the currently unidentified mechanisms by which they potentially mediate their pathogenic effects. Altogether, accumulating evidence supporting the pathogenic role of autoantibodies implies that therapies targeting the elimination or reduction of autoantibody levels may be effective in the management of IIM.

### Role of B cells in IIM

**Figure 2.**
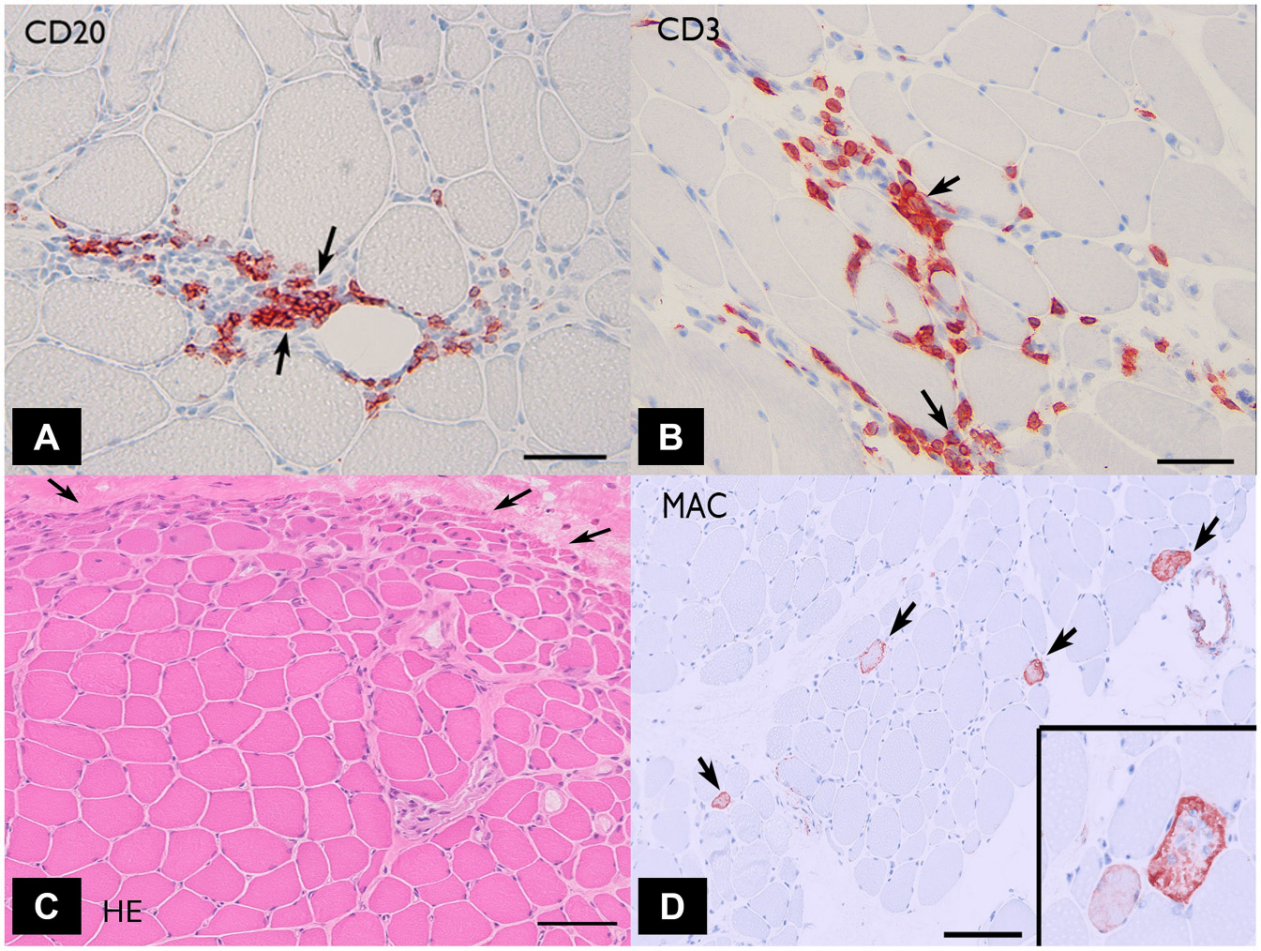
Histopathological findings in idiopathic inflammatory myopathies.

Legend: A: endomysial lymphocytic infiltrates. Arrows in A indicate CD20+ B lymphocytes Scale bars: 60 μm B: Endomysial lymphocytic infiltrates. Arrows in B indicate CD3+ T lymphocytes in IIM. Scale bars: 60 μm. C: H&E staining: perifascicular atrophy (arrows) in dermatomyositis (DM). Scale bar:120 μm. D: Membrane attack complex (MAC, complement C5b-9) immunostaining showing sarcoplasmic and sarcolemmal deposition (arrows and inset) in muscle fibers from a case of immune-mediated necrotizing myopathy (IMNM). Scale bar: 120 μm.

B cells are key regulators of the adaptive immune system. Important functions include presenting antigens to T cells, producing antibodies that recognize antigens on the cell surface of target cells, leading to cell death, and releasing cytokines enhancing pro-/anti-inflammatory processes. B cells are implicated as major drivers of immunopathogenesis in IIM. This is substantiated by the presence of autoantibodies in patients with IIM, as well as the presence of autoantibody-producing B cells and plasma cells in muscle biopsies (Figure 2A).^32,56^ Moreover, patients with active disease show elevated levels of activated B cells in peripheral blood and increased serum levels of B-cell activating factor (BAFF). In addition, evidence indicates that B cells may locally mature into plasma cells within muscle tissue.^57,58^ The role of B cells in IIM pathogenesis is further underscored by the therapeutic benefits observed with rituximab, a B-cell depleting agent.^59^ Rituximab is a monoclonal antibody targeting CD20-expressing B cells (Figure 1), resulting in their elimination primarily via antibody-dependent cellular toxicity (ADCC). Nevertheless, only 65% of IIM patients show complete or partial remission on this therapy.^60^ This limited efficacy may be attributed to rituximab's inability to fully deplete tissue-resident (autoreactive) B cells and plasma cells in the bone marrow, muscle tissue and lymph nodes, as CD20 is only partially expressed on plasmablasts and absent on plasma cells. Furthermore, the depletion of tissue-resident B cells is likely not achieved due to the poor tissue penetration of rituximab and/or reduced local availability of immune cells required for ADCC-mediated clearance.^61,62^ As a result, these autoreactive B cells remain active and continue to produce autoantibodies, thereby contributing to persistent disease activity. This highlights an unmet need for B-cell targeting treatment strategies capable to overcome these limitations. In this regard, CAR T-cell therapy or JAK/STAT inhibitors represent promising approaches, which will be discussed below.

### Role of T cells in IIM

T cells are essential components of the adaptive immune system, primarily comprising CD4 + and CD8+ T cells. Effector CD4 + T cells have a variety of functions, including the activation of B cells, thereby promoting their proliferation, differentiation, and antibody production. They also facilitate the activation of macrophages and cytotoxic CD8+ T cells, which in turn eliminate target cells, and enhance the production of inflammatory cytokines such as IFNs.^63^ Autoreactive T cells have been identified as key contributors of immunopathogenesis in autoimmune diseases such as rheumatoid arthritis (RA) and vasculitis, and their role is also implicated in IIM.^63^ In muscle biopsies from IIM patients, T cell infiltrates (often more numerous than B cell infiltrates) have been detected (Figure 2B). Effector molecules released by CD8+ T cell, such as perforins and granzymes (Figure 1) may contribute to myofiber damage and promote chemotaxis.^1,56,64,65^ In DM, perivascular T cells are mainly composed of CD4+ T cells, while in ASyS, the endomysial infiltrates are mainly composed of CD8+ T cells.^66,67^ In muscle tissue of IIM, the expression of various regulatory and costimulatory molecules on T cells, including cytotoxic T-lymphocyte-associated protein 4 (CTLA-4) and CD28 is upregulated, leading to impaired and dysregulated T cell function. A recent study using T cell receptor (TCR) profiling identified clonal T cell expansion in muscle tissue, with an increase in clonal expansion correlating with higher disease activity. Additionally, identical expanded clonal T cells were present in both muscle tissue and peripheral blood of IIM patients, suggesting that these clones may be autoreactive T cells.^68^ Expanded T cell clones persisted in a subset of refractory patients before and after treatment, indicating their potential involvement in disease chronicity.^69^

In IBM, CD8+ T cells are present in the muscle tissue, where they surround and invade the muscle tissue, suggesting cytotoxic T cell-mediated muscle damage.^70^ In line with this, using a novel PET-tracer, cytotoxic T cell populations could be visualized in the limb musculature of patients with IBM.^71^ These cytotoxic T cells are clonally expanded and shared between blood and muscle tissue in IBM patients.^72–74^ Several studies have shown that the muscle-infiltrating T cells in IBM mainly consist of highly differentiated T cells, which produce high levels of perforin and granzyme. The presence of terminally differentiated T cell populations may account for the lack of response to immunosuppressive treatment in IBM, as previous research indicates that these cells are not effectively modulated by such therapies and may even expand, possibly due to resistance to mechanisms mediating cell death.^17,75,76^ Altogether, this evidence highlights the role for T cells in IIM and suggests that targeting T cells may provide a promising therapeutic approach for IIM.

### Neonatal Fc receptor

Several studies have highlighted the significant role of the FcRn receptor in the pathogenesis of autoimmune diseases.^77^ FcRn is present on various cell types, including macrophages/monocytes and endothelial cells where it binds to IgG antibody-antigen complexes, preventing the elimination of IgG antibodies through a recycling mechanism (Figure 1). In autoimmune diseases, this results in an extended half-life of circulating IgG autoantibodies, thereby promoting autoantibody-related disease activity. Moreover, FcRn receptors may further contribute to autoimmunity by stimulating immune effector functions, such as opsonization. FcRn receptor blocking has demonstrated promising results in other autoimmune-mediated neuromuscular disorders, i.e., myasthenia gravis (MG) and chronic inflammatory demyelinating polyradiculoneuropathy (CIDP).^78,79^ Consequently, FcRn receptor blocking may also have therapeutic potential in IIM, which will be discussed below.

### Interferon pathway

IFNs constitute a group of cytokines that play a pivotal role in the pathogenesis of autoimmune diseases, including RA, SLE, and particularly in IIM. Three classes of IFNs have been discovered: type I, type II and type III. Type I IFNs include several subtypes, with IFN-α and IFN-β being predominant in IIM pathogenesis. In contrast, type II IFNs consist of a single subtype: IFNγ. Both type I and type II IFNs have various functions and induce their effects primarily via the JAK/STAT signaling pathway (Figure 1). This pathway is activated when IFNs bind to cell surface receptors, leading to the downstream expression of target genes. IFNs are crucial in both initiating and sustaining disease manifestations in IIM. In DM, a predominant type I IFN signature has been identified, characterized by the upregulation of type I IFN-inducible genes in muscle, skin, and peripheral blood.^80–83^ Type I IFNs contribute to muscle damage in DM by promoting the expression of target genes that drive muscle atrophy and disrupt the vascular network, including type I IFN-stimulated gene 15 (ISG15) and by facilitating chemotaxis into the inflamed muscle tissue. Furthermore, IFN-inducible proteins are overexpressed in muscle and skin biopsy specimens from DM patients, particularly Myxovirus resistance protein (MxA).^84^ This protein is a distinctive marker of DM in muscle biopsies and is already present in the early stages of the disease, implicating an important role in the pathogenesis. The overproduction of these proteins may directly impair myofiber function: a study demonstrated that when human skeletal muscle cells were exposed to type I IFNs, they displayed a molecular profile similar to that seen in DM muscle biopsies, accompanied with production of IFN-inducible proteins.^85^ The upregulation of IFN-inducible proteins possibly may damage capillaries and myofibers, resulting in capillary damage and perifascicular atrophy (PFA) (Figure 2C), which are characteristic features in DM muscle biopsy.^86^ The IFN pathway has also been implicated in the pathogenesis of anti-MDA5 DM, as anti-MDA5 autoantibodies increase the transcription of type I IFN. This in turn promotes the differentiation of B cells into plasma cells that produce autoantibodies, including anti-MDA5 antibodies. These antibodies might form immune complexes that trigger further IFN production, thereby perpetuating a self-amplifying cycle of immune activation. Although immune complexes have not yet been demonstrated in DM muscle biopsies. While DM is defined by a type I IFN signature, ASyS seems to be characterized by a type II IFN signature, which is upregulated in the muscle, blood and lungs of these patients.^83^ Additionally, elevated levels of serological markers possibly reflecting the activation of the type I IFN pathway, such as Siglec-1, are associated with disease activity and the clinical response to treatment, in particular in DM.^87,88^

Mitochondrial abnormalities have been observed in an IIM murine model, and therapies targeting IFNγ reduced these mitochondrial dysfunctions. In this line, in IIM patients an association between IFNγ and mitochondrial abnormalities was found by molecular analysis.^89^

For IBM also an important role for IFNs in the pathogenesis is proposed, as cytotoxic T cells in IBM produce IFNγ and myofibers infiltrated by these cells exhibit an IFNγ signature.^83,90^ Moreover, previous studies revealed upregulation of IFN-inducible genes in muscle tissue and blood of IBM patients.^83^ More recently, the upregulation of IFNγ was associated with muscle atrophy in IBM muscle biopsies in mice and targeting this pathway in vivo ameliorated myofiber size.^91^ In summary, IFNs play a key role in the pathogenesis of certain types of IIM and thereby targeting IFN might have beneficial effects in IIM.^83,92,93^

### Complement system

The complement system plays a critical role in innate and adaptive immunity, and its dysregulation is implicated in various autoimmune diseases, including IIM. The complement system comprises of several proteins, including C2, C3, C5, which facilitate key immunological processes. The complement system is primarily activated through the classical pathway and alternative pathway. Although these pathways vary in the way they are activated, activation of the complement cascade via the classical or alternative pathway results in the formation of the membrane attack complex (MAC), which inserts into the membrane of target cells and induces lysis (Figure 1). Furthermore, complement proteins such as C3b promote opsonization, while others like C5a act as potent chemoattractants, recruiting immune cells to sites of inflammation.^94–96^ The role of complement derangements in IIM pathogenesis is implicated by the presence of complement proteins deposits and MAC in muscle tissue and endomysial capillaries.^97,98^ For decades, DM has been viewed as a complement-dependent microangiopathy, in which the endothelium of capillaries is chronically attacked by MAC, serving as the primary target of the disease. According to this concept, autoantibodies against endothelium of capillaries are thought to activate the complement system in DM, resulting in the formation of MAC.^99–101^ Following MAC activation, pro-inflammatory cytokines are released and migration of other immune cells into the endomysium is promoted.^102^ These inflammatory processes sequentially cause endothelial cell swelling, capillary necrosis, and perivascular inflammation, leading to a reduction in the density of endomysial capillaries and endothelial cell necrosis in muscle tissue. According to this hypothesis, eventually, these processes result in PFA which is highly characteristic of DM in muscle biopsies.^97,102–104^ However, whether PFA and muscle fiber injury occurs as a result of complement activation is not fully defined, as another hypothesis proposes that these may result from the overexpression of type I IFN-inducible genes. MAC accumulation is already present in the endomysial capillaries at an early stage of DM, even before noticeable damage to muscle tissue or capillaries occurs, supporting its early role in IIM pathogenesis and possibly as an initiating event.^103,105^ While the presence of complement proteins and MAC deposits in muscle tissue is well recognized, uncertainties remain whether the complement pathway is the primary driver of pathogenesis in DM or merely a secondary response to other immunological processes that predominantly drive the disease.^104^ The mechanism by which the complement pathway is activated and its role in endothelial tissue injury and PFA in DM are not fully characterized yet. Several mechanisms have been proposed for complement activation in DM, including complement activation by antibody binding to endothelial antigens or antibody-independent complement activation in response to endothelial tissue damage.^104^ One study suggested that the complement pathway may be activated in an antibody-independent manner through the direct binding of complement protein C1q to damaged capillaries.^81,86,106^ The important role of the complement system in DM is further supported by beneficial effects of intravenous immunoglobulins (IVIg), as one of its mechanisms of action involves the inhibition of complement proteins, including C3b and thereby blocking MAC formation.^97,107–109^

In IMNM, MAC deposits are present in muscle biopsies (Figure 2D) and it has been suggested that autoantibody-dependent complement activation contributes to muscle damage. Previous studies demonstrated that the pathogenic effects of IMNM autoantibodies both *in vitro* and *in vivo*, were enhanced by the presence of an intact complement system.^42,44^ Another study showed MAC accumulation in muscle fibers along with C1 deposits in patients with autoantibody positive IMNM, suggesting its role in myofiber necrosis and direct activation of the complement pathway by autoantibodies.^110^ These findings indicate the likely pathogenic role of anti-SRP and anti-HMGCR autoantibodies and their ability to activate the complement system.^44^

Altogether these data may implicate a key role for the complement system in both DM and IMNM and thereby complement inhibitors might have therapeutic potential.

## Current therapies in IIM

The management of IIM remains challenging due to the disease's heterogeneity and involvement of extramuscular manifestations. These factors, along with the rare occurrence, lack of standardised internationally accepted outcome measures and refined international disease classification criteria, make it difficult to conduct high-quality large clinical trials.^111,112^ Although significant advances have been made in the development of disease classification criteria in recent years with more refined criteria for IMNM, DM and ASyS. Some of the outcome measures used in clinical trials for IIM include the Cutaneous Dermatomyositis Disease Area and Severity Index (CDASI), the International Myositis Assessment & Clinical Studies Group (IMACS) core set measures, which include manual muscle testing (MMT) on a 0–10 point scale, Definition of Improvement (DOI), and Total Improvement Score (TIS). These outcome measures are described extensively elsewhere.^111^ Treatment of IIM is often empirical and based on expert opinion and there are no standardised consensus-based international guidelines.^113^ There is insufficient evidence on current treatment options and evidence for their efficacy are derived from retrospective cohort studies and case series. Only for IVIg robust evidence is available with some evidence on rituximab, which will be described below.^113–115^

Except for IBM, conventional treatment of IIM consists of immunosuppressive therapy with glucocorticoids and steroid-sparing agents (e.g., azathioprine, methotrexate) as first-line therapy. Second-line treatment includes mycophenolate mofetil or calcineurin inhibitors (tacrolimus, ciclosporine) and IVIg. Third-line therapy includes rituximab or cyclophosphamide, although rituximab is increasingly used in early phases of the disease.^1,114^ Special therapeutic considerations are important in IIM regarding to subtype or extramuscular manifestations. In IMNM patients, a more intensive treatment approach in the early disease stage with administration of IVIG or rituximab is advocated.^114,116,117^ In the presence of moderate to severe ILD, mycophenolate mofetil is often preferred as first-line treatment.^118^ For severe or rapidly progressive IIM-associated ILD, rituximab, cyclophosphamide or calcineurin inhibitors should be considered.^119–124^

The Rituximab in Myositis (RIM) study investigated the efficacy of rituximab in a prospective, double-blind, randomized, delayed-start design. Although the study failed to meet its primary endpoint, which was the difference in time to DOI, 83% of the patients met the DOI with a median time of 20 weeks.^125^ A systematic review showed that 65% of IIM patients respond to rituximab, with 45% achieving a complete response. The overall efficacy of rituximab for muscle, lung and skin manifestations was 62%, 68% and 62%, respectively. In these studies most of the patients received rituximab due to refractory disease or ILD.^60^ In a recent study, rituximab was administered as first-line therapy in treatment-naive myositis patients and all patients showed improvement in muscle strength at a 6-month follow-up.^126^

IVIg, the only FDA/EMA approved treatment for IIM, has shown beneficial effects in refractory DM in two previous double-blind placebo-controlled trials and has also shown clinical improvement in other IIM subtypes, such as IMNM, in retrospective studies.^109,127,128^ Currently IVIg is not administered as first-line or induction therapy except for severely affected patients, however previous studies have shown that IVIg monotherapy as first-line treatment may also be beneficial in the early phase of the disease.^116,129^ Given these encouraging results in both newly diagnosed IIM and refractory patients, there are trials underway to assess the efficacy of early add-on IVIg treatment in IIM (Table 2).

**Table 2.** New and emerging therapies in idiopathic inflammatory myopathies (IIM) and ongoing clinical trials.

Drug name	Mechanism of action	Route	Studies	Disease subtype	Results	Region	Publication/ ClinicalTrials.gov ID
**B-CELL DEPLETING THERAPIES**							
Daratumumab	Target CD38, resulting in B cell depletion	IV	Case reports	DM, IMNM	Clinical symptoms improved; improvement muscle weakness; improvement ILD; disease remission	Germany (2x), Singapore, Canada	Holzer^130^, Chua^131^, Ostendorf^132^, Landon-Cardinal^133^
CD19 CAR T-cell therapy	Target CD19, resulting in B cell depletion	IV	Case reports	ASyS	Clinical symptoms improved; improvement in muscle strength, improvement of extramuscular symptoms including ILD; drug-free remission	Germany (3x)	Pecher^134^, Müller^135^, Taubmann^136^
			Case series	ASyS	Clinical symptoms improved; normalization in muscle strength and cessation of extramuscular disease activity, including ILD; drug-free remission	Germany	Müller et al.^137^
			Case report	IMNM	Clinical symptoms improved: improvement in muscle strength, drug-free remission	USA, China	Volkov^138^, Wang^139^
BCMA CAR T-cell therapy	Target BCMA, resulting in B cell depletion	IV	Case report	IMNM, ASyS	Clinical symptoms improved: improvement in muscle strength, drug-free remission	China, Germany	Qin^140^, Müller et al.^141^
Teclistamab	Redirecting T cells to target BCMA B cells	SC	Case report	DM	Improvement in clinical symptoms; improvement in muscle weakness, skin manifestations and other extramuscular symptoms, including arthritis and ILD	Germany	Hagen et al.^142^
CD19 CAR T-cell therapy	Target CD19, resulting in B cell depletion	IV	Multiple phase 1 / 2 studies, single-arm open label studies and RCTs	DM/ASyS/ IMNM/PM	Pending	China (3x), international multicenter (2x), Italy (1x), Germany (1x)	NCT06665256; NCT06154252; NCT06828042; NCT06821659; NCT06685042; NCT06347718
CD19-BCMA CAR T-cell therapy	Target CD19-BCMA, resulting in B cell depletion	IV	Phase 2 study, single arm open label study	Not specified	Pending	China	NCT06794008
**COMPLEMENT INHIBITORS**							
Zilucoplan	C5 inhibitor	IV	Phase 2 study, RCT	IMNM	No clinical effect	International multicenter	Mammen et al.^143^
Eculizumab				DM	No clinical effect	Unknown	Takada et al.^144^
Empasiprubart	C2 inhibitor	IV	Phase 2 study, RCT	DM	Pending	International multicenter	NCT06284954
Ravulizumab	C5 inhibitor	IV	Phase 2 / 3 study	DM	Terminated early, primary endpoint not met		NCT04999020
Efgartigimod	Targeting FcRN, prevention recycling IgG	IV	Open label single arm study	IMNM	Modest clinical improvement, particularly in CK levels	China	Yang et al.^145^
**FcRn inhibitors**							
Efgartigimod	Targeting FcRN, prevention of IgG recycling	SC	Phase 2 /3 study, RCT	DM, IMNM, ASyS	Pending	International multicenter	NCT05523167
Nipocalimab		IV	Phase 2 study, RCT				NCT05379634
**IFN INHIBITORS**							
Dazikubart	IFNβ	IV	Phase 2 study, RCT	DM	Improvement in especially skin disease activity and also muscle disease activity	International multicenter	Fiorentino et al.^146^
Dazikubart	IFNβ	IV	Phase 3 study, RCT	DM, ASyS, OM	Pending	International multicenter	NCT05895786
Anifrolumab	Targeting IFNAR1	SC	Phase 3 study, RCT		Pending		NCT06455449
Daxdilimab	Targeting IFN production		Phase 2, RCT		Pending		NCT05669014
**JAK INHIBITORS**							
Tofacitinib, baricitinib, ruxolitinib	Targeting Janus kinase pathway, blocking IFN signalling	PO	Open label studies single-arm, systematic review	DM	Improvement in disease activity, including in skin disease activity, muscle disease activity and ILD	NA	Paik et al.^147^
Tofacitinib	Targeting Janus kinase pathway, blocking IFN signalling	PO	Small case series	ASyS	Improvement in ILD, muscle disease and arthritis	China	Shan et al.^148^
Filgotinib	Targeting Janus kinase pathway, blocking IFN signalling	PO	Phase 2 study, single-arm open label	DM/ASyS	Pending	The Netherlands	NCT06285539
Baricitinib	Targeting Janus kinase pathway, blocking IFN signalling	PO	2 RCTs: phase 2 and phase 3	DM, ASyS, OM	Pending	UK (1x), France (1x)	NCT04208464; NCT04972760
Ruxolitinib			Phase 2/3 study, RCT	IBM	Pending	France	NCT06536166
**JAK1/TYK2 INHIBITORS**							
Brepocinitib	Targeting JAK1/TYK2, blocking IFN signalling	PO	Phase 3 study, RCT	DM	Pending	International multicenter	NCT05437263
Brepocinitib			Phase 2 study, single-arm open label	DM	Pending	USA	NCT06433999
**TYK2 INHIBITORS**							
Nintedanib	Targeting TYK2, blocking IFN signalling	PO	Phase 4 study, RCT	DM, ASyS, OM	Pending	USA	NCT05799755
GLPG3667			Phase 2 study, RCT	DM	Pending	International multicenter	NCT05695950
**T-CELL TARGETING THERAPIES**							
Abatacept	Interfering with co-stimulatory signal of T cells, blocking T cell proliferation and effector function	IV	Phase 3 study, RCT	DM/ASyS/IMNM	Possibly beneficial in non-DM patients as add-on therapy	International multicenter	Aggarwal et al.^149^
Sirolimus	Blocking T cell activation and proliferation	PO	Phase 2 study, RCT	IBM	No change in maximal voluntary isometric knee extension strength after 12 months, improvement in secondary outcomes	France	Benveniste et al.^150^
Abatacept	Interfering with co-stimulatory signal of T cells, blocking T cell proliferation and effector function	IV	Phase 2 study, RCT	ASyS	Primary endpoint not met; no improvement in pulmonary symptoms. Trend towards improvement in pulmonary parameters on longterm	USA	Aggarwal et al.^151^
Sirolimus	Blocking T cell activation and proliferation	IV	Phase 3 trial, RCT	IBM	Pending	USA, Australia	NCT04789070
ABC008	Targeting KLRG1, highly differentiated cytotoxic T cells	IV	Phase 2 / 3 trial, RCT	IBM	Pending	International multicenter	NCT05721573
Ulviprubart			Phase 2 trial, open label single arm study		Pending	USA, Australia	NCT06450886
**IMMUNOGLOBULINS**							
Immunoglobulins	Multiple mechanisms of action	IV	Phase 2 study RCT	DM, ASyS, IMNM, OM	Pending	The Netherlands	NCT05832034

The therapeutics in the table are not exhaustive of all existing treatment options in IIM. Tumor necrosis factor-alpha (TNF-α) inhibitors (e.g., infliximab) and B-cell activating factor (BAFF) inhibitors—(e.g., belimumab)—are not included; as earlier trials with these therapeutics were not conclusive or negative and no studies are currently underway involving these agents. Legend: ASyS: antisynthetase syndrome; BCMA: B cell maturation antigen; CAR T cell: chimeric antigen receptor T cell; DM: dermatomyositis; FcRn: neonatal Fc receptor; IBM: inclusion body myositis; IFN: interferon; IFNAR1: type I IFN receptor; IFNβ: interferon β; ILD: interstitial lung disease; IMNM: immune-mediated necrotizing myopathy; IgG: immunoglobulin G; IV: intravenous; JAK: Janus kinase; KLRG1: killer cell lectin-like receptor G1; NA: not applicable; OM: overlapmyositis; PM: polymyositis; PO: per os; RCT: randomized controlled trial; SC: subcutaneous; TYK2: tyrosine kinase 2; UK: United Kingdom; USA: United States of America.

No pharmacological treatment is currently available for IBM. The efficacy of IVIg in IBM is debated; it has shown mild to moderate improvement in some studies with a low level of evidence.^17^

## Emerging therapies in IIM

### B-cell targeting therapies, CAR T-cell therapy and T-cell engager therapy

Recently, CD19-targeting CAR T-cell therapy has gained substantial attention in the treatment of B cell-driven autoimmune diseases, including IIM, to target (autoreactive) B cells (Figure 1). In CAR T-cell therapy, patient T cells are isolated, genetically engineered in the laboratory to express a CAR directed against a specific antigen, such as CD19 on B cells (Figure 1). After lymphodepleting chemotherapy, the engineered CAR T cells are infused back into the patient to selectively eliminate target cells as a living drug. CAR T-cell therapy has been successfully applied in IIM patients, as demonstrated by several case reports and small case series. For instance, in four severely affected patients with autoantibody-positive ASyS (anti-Jo-1 or anti-PL7), with ILD and multi-organ involvement who had previously failed several immunosuppressive drugs, CD19-targeted CAR T-cell therapy resulted in rapid clinical improvement of muscle and pulmonary symptoms, inducing drug-free remission during follow-up period of up to one year.^134–137^ CAR T-cell therapy has also resulted in improvement of clinical symptoms in three patients with autoantibody-positive refractory IMNM, which were maintained over a follow-up period of up to 18 months.^138–140^ All patients treated with CAR T-cell therapy showed rapid B cell depletion. Unlike rituximab, there was no long-term B cell depletion, and repopulation of B cells occurred around 100 days after CAR T cell infusion. The repopulating B cells were predominantly naive and memory B cells, whereas plasmablasts being either low in number or not detectable. Remarkably, even after B cell repopulation, no relapses were observed. Additionally, in contrast to rituximab, CAR T-cell therapy resulted in the depletion of tissue-resident B cells in lymph nodes.^152^ Collectively, these findings imply that CAR T-cell therapy potentially leads to the elimination of autoreactive B cells and may induce a functional reset of the immune system in patients with B cell-mediated autoimmune diseases, potentially even curing the disease. However, larger trials are necessary to fully elucidate its therapeutic efficacy and safety (ongoing CAR T trials in IIM are listed in Table 2).

CAR T-cell therapy has been generally well tolerated in patients with autoimmune diseases, with relatively limited adverse events. It is important to note that the treatment is intensive and may be associated with severe toxicities, including cytokine release syndrome (CRS), immune effector cell-associated neurotoxicity syndrome (ICANS) and infections. Therefore, careful patient selection, thorough pre-screening and vigilant monitoring during and after treatment are essential. To date, no high-grade CRS or ICANS, nor serious infections have been reported in patients receiving CAR T-cell therapy for autoimmune diseases.

Additionally, concerns have been raised regarding the potential increased risk of secondary malignancies, such as T-cell malignancies and myelodysplastic syndrome/acute myeloid leukemia, following CAR T-cell therapy in hematological patients. However, these incidences are not higher than expected, given that all hematological patients involved had significant prior chemotherapy exposure, which itself already increases the risk of secondary malignancies. The exact contribution of CAR T-cell therapy in the development of these secondary malignancies remains unclear.^153–155^ In summary, the side effects of CAR T-cell therapy in patients with autoimmune diseases seems mild and manageable, although the long-term effects remain to be determined.

B lineage cells can also be targeted using other approaches, such as antibodies or T-cell engagers (TCE). Daratumumab, a monoclonal antibody targeting CD38 on mature B cells (Figure 1), has shown beneficial effects in case reports involving severely affected patients with anti-MDA5 DM and anti-SRP IMNM.^130–133^ A small number of other monoclonal antibodies, such as obinutuzumab and ocrelizumab, have been explored for the treatment of IIM patients in case reports/case series which is described elsewhere.^156^

Recently, a TCE therapy with teclistamab has been tested in a patient with IIM. Originally developed for the treatment of malignant B cells, teclistamab redirects T cells to target B cells expressing B cell maturation antigen (BCMA), which is expressed on differentiated mature B cells. Therapy with teclistamab eventually leads to the elimination of these cells. In a patient with anti-MDA5 DM teclistamab led to improvement in muscle symptoms, skin disease and ILD.^142^

### T-cell-targeting therapies

Targeting T cells with abatacept have demonstrated efficacy in the management of autoimmune diseases and has received FDA approval for treatment of RA and psoriatic arthritis.^157,158^ Given the central role of T cells in the pathogenesis of IIM, targeting T cells has also been proposed as a therapeutic strategy in IIM. Abatacept is a fusion protein composed of the Fc region of human IgG1 linked to the extracellular domain of CTLA-4. Abatacept binds to CD80 and CD86 on antigen-presenting cells and hinders its binding to CD28, thereby preventing costimulation and thus interfering with T cell proliferation and effector function (Figure 1). Abatacept improved muscle strength in 42% of patients with lower disease activity after 6 months.^159^ While, a randomized phase 2b pilot study did not meet its primary endpoint, post-hoc analysis suggested benefits in certain subgroups (e.g., non-DM). The British Society of Rheumatology recommends considering abatacept for refractory IIM. Recently in a small randomized controlled trial (RCT), no improvement in forced vital capacity (FVC) was observed in abatacept treated patients with ASyS associated ILD at week 24, although non-statistically significant improvements in FVC were seen at week 48.^151^

Agents targeting T cells have also emerged as a novel treatment option in IBM. Sirolimus is a small-molecule inhibitor of the mammalian target of the rapamycin (mTOR) pathway and interferes with intracellular signaling, which results in inhibition of T cell activation and proliferation (Figure 1). A double-blind RCT evaluating the efficacy of sirolimus in IBM patients did not meet its primary endpoint, which was the change in maximal voluntary isometric knee extension strength after 12 months.^150^ However beneficial effects in secondary outcomes were observed, including walking distance and forced vital capacity, justifying a currently ongoing phase 3 RCT to further explore the efficacy of sirolimus (Table 2).

Several trials in IBM are investigating compounds targeting killer cell lectin-like receptor G1 (KLRG1), a marker expressed on highly differentiated cytotoxic T cells, with the aim of depleting these T cell populations (Table 2).

### Neonatal FcR inhibitors

In recent years, FcRn receptor inhibitors have emerged as a promising therapeutic approach in autoimmune diseases due to their ability to reduce (pathogenic) autoantibody levels (Figure 1).The therapeutic use of FcRn inhibitors was first investigated in MG, resulting in meaningful clinical improvement and reduction of pathogenic autoantibody levels.^78,160^ Similarly in CIDP, FcRn inhibitors reduced pathogenic autoantibody levels and demonstrated clinical benefit.^79^

The FcRn inhibitor efgartigimod was tested in a humanized mouse model of IMNM, leading to a decrease of IgG and autoantibody levels in both serum and muscle, resulting in reduced myofiber necrosis and promoting muscle fiber regeneration.^161^ A recent open-label pilot study in seven patients with refractory autoantibody-positive IMNM treated with intravenous efgartigimod showed modest clinical improvement, particularly in CK levels and mildly in muscle strength in four of the seven patients within 4 weeks, which was maintained after 8 weeks.^145^ Efgartigimod was generally well tolerated, with limited adverse events. Currently phase 2/3 RCTs are investigating the efficacy and safety of FcRn inhibitors, efgartigimod and nipocalimab, in patients with DM, IMNM and ASyS (Table 1).

### Interferon targeting therapies

Treatment with anifrolumab, a monoclonal antibody targeting type 1 IFN receptor (Figure 1), has resulted in clinical improvement in SLE patients in two phase 3 RCTs, leading to FDA approval.^162,163^ Given the central role of IFNs in IIM pathogenesis, IFN-targeting therapies are also investigated for their therapeutic potential in IIM.^83,93^ A recent multicenter, double-blind placebo-controlled RCT investigated the efficacy and safety of dazukibart, a monoclonal antibody that binds to IFNβ and inhibits signaling through the IFNα/β receptor (Figure 1).^146^ In this trial, patients with skin- and muscle-predominant DM were enrolled in different stages. In the skin-predominant cohort, at least 96% of patients receiving dazukibart met the primary efficacy endpoint, a clinically meaningful decrease in the CDASI score at week 12. Improvement in skin symptoms were sustained after drug discontinuation.

In the muscle-predominant cohort, the active treatment group showed non-statistically significant improvement of TIS and muscle strength at week 12. Dazukibart was generally well tolerated, with mild side effects reported, including upper respiratory tract infections, sinusitis, and COVID-19. Ongoing RCTs are currently investigating the efficacy and safety of targeting type I IFN in patients with DM, ASyS and OM (Table 2).

### JAK/STAT inhibitors

JAK/STAT inhibitors have been established in the treatment of various autoimmune diseases, including RA, ankylosing spondylitis and psoriatic arthritis and have shown potential as a novel treatment option in IIM to target IFNs and/or (autoreactive) B cells.^164^ In the first prospective open-label study in refractory skin-predominant DM patients, treatment with the JAK/STAT pathway inhibitor tofacitinib resulted in clinical response in all patients, including significant improvement in skin disease activity after 12 weeks.^165^ Improvement in skin symptoms were already evident after 4 weeks and were maintained up to 96 weeks.^165,166^ Subsequently, multiple small case series and open-label trials have been published assessing the efficacy and safety of various JAK inhibitors. In a prospective open-label trial, tofacitinib was administered to newly diagnosed, treatment-naive patients with DM and anti-MDA5 autoantibodies, resulting in clinical improvement in muscle weakness and skin rash in 71.4% of patients.^167^ In a retrospective study of refractory IIM patients with DM or PM, treatment with tofacitinib led to improvement in cutaneous disease activity, however no clinically relevant improvement in muscle strength was observed.^168^ Among adult refractory DM patients, according to a systematic review, treatment with a JAK inhibitor (e.g., tofacitinib and baricitinib) resulted in improvement in both skin disease activity and muscle disease activity.^147^ In patients with juvenile dermatomyositis (JDM), JAK inhibitors were effective in 95% of the patients with refractory skin manifestations and 83% of patients with refractory muscle disease.^147^ Recently, a retrospective cohort study indicated that the JAK inhibitor baricitinib can maintain efficacy in treating skin manifestations and muscle symptoms for up to 36 months in severe JDM.^169^ A detailed discussion on the treatment of JDM is beyond the scope of this review.

JAK inhibitors have also been tested in a small open-label study involving 20 patients with refractory ASyS with ILD, and demonstrated beneficial effects in clinical manifestations in 70% of the patients, including ILD, skin rash and muscle symptoms.^148^

JAK inhibitors have generally been well tolerated and have an acceptable safety profile in IIM studies. However, their use requires careful consideration, including adequate screening before starting treatment and regular monitoring during therapy, as they may be associated with an increased risk of infections, cardiovascular disease and malignancies. Importantly, a prior study in RA found that these adverse events are almost exclusively seen in a high-risk patient population, defined as those aged ≥65 years or ever-smokers, and rarely in patients without one of these risk factors.^170^ Therefore JAK inhibitors should preferably not be used in this high-risk population.

In conclusion, treatment with JAK inhibitors in refractory DM patients has resulted in significant clinical improvement, particularly in skin symptoms. In ASyS, small case series have shown beneficial effects of JAK inhibitors with an acceptable safety profile.

Several ongoing RCTs and single-arm open-label trials are investigating the clinical efficacy and safety of JAK inhibitors in IIM (Table 2). Baricitinib, a selective JAK1 inhibitor, was assessed in a RCT involving patients with DM, ASyS and OM. While definitive results are still pending, preliminary results show beneficial clinical response at 12 and 24 weeks.^171^ Table 1 shows trials with brepocitinib (targets selectively the JAK isoforms JAK1 and tyrosine kinase 2 (TYK2)) in DM and ruxolitinib in IBM.

### Complement inhibitors

Blocking complement-mediated pathogenic effects of autoantibodies with complement inhibitors has shown favourable clinical effects in autoantibody positive MG.^172^ Based on the hypothesis that activation of complement pathway has a role in disease pathogenesis in IIM as well, complement inhibitors have been proposed as a novel therapeutic approach in IIM.^44,106,110^ The IMNM-01 study tested zilucoplan, a synthetic peptide inhibitor of complement protein C5.^143^ Zilucoplan binds to C5, inhibiting its cleavage into C5a and C5b, and also binds to a specific site on C5, sterically blocking its interaction with C6, thereby preventing MAC assembly (Figure 1). This was a double-blind, phase 2 RCT that investigated the efficacy and safety of zilucoplan in autoantibody-positive IMNM patients over 8 weeks. Zilucoplan resulted in complement pathway inhibition in the active treatment group. However, there were no significant or clinically relevant changes in creatine kinase levels at week 8. Likewise, no clinically relevant or significant differences were observed in secondary clinical endpoints, including minimal response based on the TIS. Similar results were observed in the open-label extension phase of the study.

Despite the negative results of the IMNM-01 trial, the therapeutic potential of complement inhibition is not fully excluded, as there are caveats in the IMNM-01 trial design and important considerations regarding the role of complement in the immunopathogenesis of IIM. Firstly, all participants in the trial had a relatively long disease duration, and patients in the active treatment group had a mean disease duration that was 14.1 months longer than the placebo group. This seems relevant as the complement pathway may primarily have a significant role in the onset of IMNM rather than in the chronic phase of the disease.^173^ An experimental study in a humanized mouse model of IMNM showed that zilucoplan effectively prevented disease onset, but did not improve muscle strength following disease onset.^174^ This implies that complement inhibition may be more beneficial in the early stages of IMNM. Secondly, it is possible that IMNM autoantibodies contribute to disease pathogenesis or progression through non-complement-mediated mechanisms. An *in vitro* study demonstrated that anti-HMGCR and anti-SRP antibodies induced muscle atrophy and impaired muscle fiber regeneration via complement-independent mechanisms.^43^

Complement inhibitors have also been tested in DM. In a pilot study involving 13 patients with DM treatment with eculizumab, a C5 inhibitor (Figure 1), showed improvement in cutaneous scores and muscle strength in 37% and 9% of participants, respectively, although published data are very limited of this study.^144^ In DM, a multicenter RCT evaluating the efficacy of ravulizumab, another C5 inhibitor (Figure 1), was terminated early after failing to meet its primary endpoint (Table 2). Notably, inhibiting the complement pathway at a higher level of the cascade (i.e., C2) may have more therapeutic benefit as compared to terminal complement inhibition, as this may result in disruption of other immunological processes beyond MAC formation, including opsonization and chemotaxis. Currently, an ongoing trial in DM investigates the effect of empasiprubart, a C2 inhibitor (Figure 1) which blocks the complement cascade at an earlier stage.

In conclusion, the effects of complement inhibitors in IIM have been disappointing as to date, although further studies are necessary to definitively establish the therapeutic potential.

## Conclusion and future directions

The treatment landscape of IIM is rapidly evolving, with novel targets and emerging treatments on the horizon. Over the last 5 years, there has been a remarkable increase in clinical trials, in part driven by advances in understanding disease pathogenesis. Several concerns and challenges remain with regard to trial design and the conduct of studies in IIM, including lengthy placebo arms, the use of partially or non-validated outcome measures, a lack of rigorous disease classification criteria, and the limited diversity of participants.^175^ These challenges and concerns are extensively reviewed elsewhere.^175^ The increasing number of clinical trials in IIM offers promising potential for expanding our current pharmacological treatment options. An important consideration is the cost-effectiveness of therapies. FcRn inhibitors, C5 inhibitors and IVIg, in particular, are highly costly, require repeated administrations over many years (with recent insights on shorter intervals for FcRn inhibitors in MG), and appear to primarily reduce disease activity rather than achieve complete remission.^176^ On the other hand, CAR T-cell therapy, while also expensive and intensive, may result in complete, drug-free remission for an extended period with a single infusion in refractory patients.

Despite significant advances in understanding the disease pathogenesis of IIM over the past decade, the pathogenesis of IIM is still not fully understood, in particular of IBM, and further investigation is necessary to potentially identify additional therapeutic targets. One important observation is that regenerating muscle fibers express high levels of antigens, which are targeted by specific autoantibodies. While some authors propose that this expression is a physiological part of muscle regeneration, others suggest its role in persistence or even initiation of disease activity, proposing that autoimmunity is triggered in injured and regenerating muscle tissue, rather than in healthy muscle tissue.^177^ Whether the expression of autoantigens in regenerating muscle fibers has a role in disease pathogenesis remains to be elucidated, which potentially could reveal novel targets for treatment.^177–180^ In IBM, early mitochondrial abnormalities have been implicated in the pathogenesis and upregulation of IFNγ in regenerating muscle fibers has been found by recent studies, suggesting they may offer novel therapeutic targets.^31,91^ In this review, we describe the immunopathogenesis of IIM and provide an overview of recent advances in treatment of IIM and emerging therapies. Promising therapeutics in the future include IFN inhibitors, JAK inhibitors, FcRn blockers and CAR T-cell therapy, whereas the potential efficacy of complement inhibitors remains unclear. Future trials will be crucial to establish the efficacy and safety of these treatments in IIM.
